# Clinical outcomes and prognostic factors of stereotactic body radiation therapy combined with gemcitabine plus capecitabine for locally advanced unresectable pancreatic cancer

**DOI:** 10.1007/s00432-019-03066-z

**Published:** 2019-10-30

**Authors:** Ze-Tian Shen, Han Zhou, Ao-Mei Li, Xiao-Qin Ji, Chang-Chen Jiang, Xi Yuan, Bing Li, Xi-Xu Zhu, Gui-Chun Huang

**Affiliations:** 1 Department of Radiation Oncology, Jinling Hospital, Medical School of Nanjing University, Nanjing, 210002 Jiangsu China; 2 Department of Medical Oncology, Jinling Hospital, Medical School of Nanjing University, Nanjing, 210002 Jiangsu China

**Keywords:** Locally advanced pancreatic cancer, Stereotactic body radiation therapy, Prognostic factors, CyberKnife

## Abstract

**Purpose:**

This study aimed to evaluate the clinical outcomes, toxicity, and prognostic factors of SBRT combined with gemcitabine plus capecitabine (GEM-CAP) in treating locally advanced pancreatic cancer (LAPC).

**Methods:**

A total of 56 patients with LAPC treated with SBRT combined with GEM-CAP were reviewed from October 2010 to October 2016. The median total prescription dose at five fractions was 40 Gy (30–50 Gy). The patients were subjected to two cycles of GEM-CAP before SBRT. GEM-CAP chemotherapy was then offered for four cycles or until disease tolerance or progression. The primary endpoints included overall survival (OS) and progression-free survival (PFS).

**Results:**

The median OS and PFS from the date of diagnosis was 19 (95% CI 14.6–23.4) and 12 months (95% CI 8.34–15.66), respectively. The 1-year and 2-year survival rates were 82.1% and 35.7%, whereas the 1-year and 2-year PFS rates were 48.2% and 14.3%, respectively. The median carbohydrate antigen 19-9-determined PFS time was 11 months (95% CI 5.77–16.24). Multivariate analysis demonstrated that tumor diameter, lymph node metastasis, pre-treatment CA19-9 level, and post-treatment CA19-9 decline were independent prognostic factors (*p* < 0.05). Acute toxicity was minimal, with two cases (3.6%) experiencing grade 3 duodenal obstruction. No adverse events greater than grade 3 occurred. In late toxicity, three patients (5.4%) developed grade 3 gastrointestinal toxicity and two (3.6%) suffered from perforation caused by grade 4 radiation enteritis and intestinal fistula.

**Conclusions:**

The combination of Cyberknife SBRT and GEM-CAP achieved excellent efficacy with acceptable toxicity for LAPC.

**Electronic supplementary material:**

The online version of this article (10.1007/s00432-019-03066-z) contains supplementary material, which is available to authorized users.

## Introduction

Pancreatic cancer is the fourth most deadly malignant tumor worldwide, showing a 5-year survival rate of < 5%. Its location is deep and hidden and patients at its early stages demonstrate no specific symptoms, thereby making early diagnosis difficult. Complete surgical resection is the best curative option. Unfortunately, most patients are unresectable at initial diagnosis, with only < 20% as surgical candidates (Siegel et al. 2016; Wolfgang et al. 2013). Induction chemotherapy followed by concurrent chemoradiotherapy is the available treatment option for inoperable locally advanced pancreatic cancer (LAPC) (version 1. 2019). However, the results of many randomized controlled clinical studies are contradictory. Concurrent chemoradiotherapy has been suggested to bring survival benefits in some clinical studies, but has been found to be non-beneficial in others (Chauffert et al. 2008; Huguet et al. 2007; Loehrer et al. 2011). In the LAP07 randomized clinical trial, locally advanced pancreatic cancer with disease controlled after 4 months of induction chemotherapy, there was no significant difference in overall survival with chemoradiotherapy compared with chemotherapy alone (Hammel et al. 2016). The main reason for this inconsistency is due to the side effects of conventional (traditional) radiotherapy techniques that offset survival benefits in these studies. So, there is no consensus on the optimal management of LAPC. Hence, induction chemotherapy followed by chemoradiation or chemotherapy alone or enrollment of clinical trials was employed based on NCCN guidelines (version 1, 2019). While in view of the shortcomings of conventional radiotherapy, stereotactic body radiation therapy (SBRT) has been a promising option in pancreatic cancer due to its inherent advantages (Dieterich et al. 2008).

As a new radiotherapy method, stereotactic body radiation therapy (SBRT) can avoid the error caused by respiratory movement and accurately track tumor movement by synchronizing with respiratory movement through synchronous respiratory tracking technology (Synchrony), accurately providing increased dose to the tumor, while reducing the dose exposed to normal tissues (Jumeau et al. 2018; Sutera et al. 2018). Treatment time is shortened and the effect is obtained rapidly. The planning target volume (PTV), as well as the volume of normal intestine and stomach irradiated by high dose, is remarkably reduced, because of the high conformity of the tumor target area in SBRT. The dose reaching the gastrointestinal tract remarkably decreases, causing decreased incidence of vomiting and nausea. Therefore, the combined application of SBRT and chemotherapy can remarkably reduce the toxicity and side effects caused by conventional concurrent chemoradiotherapy (Boone et al. 2013; Gurka et al. 2017), avoid delay or interruption of the development of adjuvant chemotherapy due to the side effects of radiotherapy, and ensure the normal progress of comprehensive treatment.

In the early 2007, a phase III trial compared the efficacy and safety of gemcitabine (Gem) plus capecitabine (GemCap) versus single-agent Gem in advanced/metastatic pancreatic cancer (Herrmann et al. 2007). A total of 319 patients were enrolled. GemCap failed to improve OS at a statistically significant level compared with standard Gem treatment. The safety of GemCap and Gem was similar. However, in the subgroup of patients with good performance status, median OS was improved significantly (10.1 vs. 7.4 months, respectively; *p* = 0.014). Cunninghamm et al. (Cunningham et al. 2009) concluded that GEM-CAP significantly improved patients with LAPC objective response rate (19.1% vs. 12.4%; *p* = 0.034) and PFS (*p* = 0.004) and was associated with a trend toward improved OS (*p* = 0.08) compared with GEM alone. Later, ESPAC-4 clinical trial (Neoptolemos et al. 2017) also showed that gemcitabine plus capecitabine regimens could improve the median OS compared with the gemcitabine alone (*p* = 0.032) for patients with resected pancreatic cancer. Furthermore, there was no considerable difference in toxic and adverse reactions. Currently, FOLFIRINOX regimen is the first-line treatment for LAPC (version 1, 2019). Combined with radiotherapy, the median OS can reach 31.4 months (Murphy et al. 2019). It is superior to the GEM-CAP regimens in terms of survival benefit. However, the toxicity and side effects were relatively serious, even if the modified regimens especially in combination with radiotherapy, which would further increase the side effects. Therefore, we retrospectively analyzed the efficacy, toxicity, and prognostic factors of SBRT combined with gemcitabine plus capecitabine (GEM-CAP) in 56 cases of patients with inoperable LAPC.

## Materials and methods

### Clinical data

From October 2010 to October 2016, 65 patients with LAPC were included in a retrospective study at the CyberKnife SBRT Center, Jinling Hospital. No patient received pancreatic radiotherapy prior to SBRT. The diagnosis of adenocarcinoma for all patients was confirmed histologically. All patients underwent chest-enhanced and abdomen-enhanced CTs to evaluate the condition. Abdominal-enhanced MRI was used to assist the diagnosis of the extent of liver metastases and pancreatic lesion invasion that could not be determined by CT. The program was confirmed by pancreatic surgery experts, pathology experts, radiotherapy experts, imaging specialists, oncologists, and gastroenterologists to determine locally advanced non-metastatic unresectable pancreatic cancer. The criteria were based on National Comprehensive Cancer Network (NCCN) guidelines (version 1, 2019). All patients were subjected to SBRT in the Department of Radiation Oncology of Jinling Hospital and signed written informed consent for treatment. This study was approved by the institutional review board.

### Chemotherapy

GEM-CAP was applied for all patients. Gemcitabine was delivered as a 1000 mg/m^2^ intravenous infusion administered once a week for 2 of every 3 weeks (one cycle). Capecitabine was administered orally for 14 days followed by 7 days of rest at a daily dose of 2500 mg/m^2^/day (1250 mg/m^2^ twice daily). Patients were restaged with CT after the second cycle. Patients without metastasis received GEM-CAP for the third cycle, during which timed fiducial placement, if required, and SBRT treatment plans were performed. SBRT was delivered during the weekoff between the third and fourth cycles of chemotherapy. Following SBRT, patients continued to receive GEM-CAP until drug tolerance, disease progression, or the completion of at least six cycles at the discretion of the treating medical oncologist. If digestive tract reaction and bone marrow suppression occurred, positive symptomatic treatment was carried out to avoid affecting the normal course of treatment.

### Positioning and target delineation

All 56 patients were treated using a CyberKnife SBRT system (Accuray Incorporated, Sunnyvale, CA, USA) via respiration synchronous tracking (Synchrony). More than three 6.0 mm × 0.8 mm markers were implanted within or around the tumor using a CT-guided 19G needle. CT scan was performed to observe whether the markers were in their proper positions 24 h after implantation. A CT scan was repeated 7 days after implantation. At this time, local hemorrhage and edema subsided around the gold seed fiducial, while it was relatively stable and immobile.

Patients assumed the supine position with the body fixed with a vacuum pad. Before positioning, the patients were allowed to fast for > 4 h. A total of 100–150 ml of positive oral contrast agents was administered at 10, 20, and 30 min before the CT scan to allow the viewing of the stomach and intestines. Spiral CT (Brilliace Big Bore 16 CT Philips Germany) scanning was conducted with a slice thickness of 1 mm. Pancreatic scanning consisted of two phases, namely, arterial and venous phases. Pancreatic scans covered 15 cm above and below the lesions. The pancreatic MRI was arranged in all patients to delineate tumor volumes. The gross target volume (GTV) and PTV were determined according to the tumor volume. We added a 2–3 mm margin to the GTV to account for the residual inaccuracy of synchrony. The prescription dose was defined as 100% of the GTV dose. The total PTV dose was > 95% of the prescription dose.

### Treatment mode and methods

When the CyberKnife SBRT treatment plan was designed, normal tissue dose around the lesion was strictly limited (Benedict et al. 2010). Supplementary Table 1 shows the limit dose standards of normal tissues. Conformity index (CI), which is the ratio of tissue volume to tumor volume wrapped by a prescription isodose line, and new CI (nCI), which corresponds to CI × tumor volume/tumor volume wrapped by prescription isodose line, were used to assess the treatment plan.

Before treatment, a respiratory monitoring device was used to continuously detect the position of the infrared generator placed on the patient’s chest to create a dynamic respiratory rhythm and X-ray digital imaging of kV level was collected at different time points of respiratory rhythm to obtain the dynamics model between gold seed fiducial (tumor) position and respiratory rhythm. Subsequently, the respiratory model was used to guide the accelerator in tracking the lesions within the pancreas and providing dynamic radiation. The prescription dose provided to the lesions was 30–50 Gy (median dose: 40 Gy) once per day at a fraction number of 5. During the treatment, symptomatic treatments for nausea, dehydration, vomiting, fatigue, anorexia, loss of appetite, and other complications were provided to the patients.

### Follow up and evaluation

Abdominal enhanced CT or MRI scan was performed 1 month after SBRT. The main contents of the review included imaging examination; routine blood, liver, and kidney function tests; carcinoembryonic antigen testing; and carbohydrate antigen 19-9 (CA19-9) testing. Changes in lesions and inflammation/tissue necrosis were observed, and short-term efficacy was evaluated. The patients were then followed up every 3 months. The primary endpoints of the study were overall survival (OS) and progression-free survival (PFS). RECIST 1.1 (Eisenhauer et al. 2009) Criteria in Solid Tumors was used to evaluate treatment efficacy. The imaging results were evaluated by two experienced radiologist directors based on the physician’s independent reading assessment. If their opinions were non-concordant, a collective reading agreement was conducted. If disagreements still remained, then the group conducted collective reading to reach a consensus. Acute and long-term toxicities were defined as adverse events occurring at < 3 months and > 3 months after SBRT, respectively. Toxicity was scored according to Common Terminology Criteria for Adverse Events version 4.0.

### Statistical analysis

The SPSS 18.0 statistical software was applied for data analysis. OS and PFS rate estimates were calculated using the Kaplan–Meier method and compared using stratified log-rank test. Multivariate analysis of survival was carried out using Cox’s regression model. PFS and OS were calculated from the date of diagnosis to the date of progression or death. A *p* value of < 0.05 indicates statistical significance.

## Results

### Patient characteristics

From October 2010 to October 2016, 9 of the 64 patients progressed with metastatic disease after two cycles of inducing chemotherapy. The remaining 56 patients received Cyberknife SBRT. After SBRT, the GEM-CAP regimen was continued for four cycles. The median age at diagnosis was 62 years (range 38–84 years). Out of the 56 patients, 51 showed elevated CA19-9 (> 37 IU/ml), 42 suffered from abdominal back pain, and 26 exhibited jaundice. One week before SBRT, total bilirubin (> 21 μmol/l) and direct bilirubin (> 10 μmol/l) were more than normal in 27 patients. Table 1 displays the patients’ demographics and baseline characteristics.

**Table 1:** Clinical data of the 56 pancreatic cancer patients before CyberKnife radiosurgery treatment

Item	Cases	Percentage (%)
Gender		
Male	36	57.1
Female	20	42.9
Age		
Median (range)	62 (38–84)	
< 60	26	46.4
≥ 60	30	53.6
ECOG scores		
0	10	17.9
1	38	67.9
2	8	14.2
The position of tumor		
Head	24	42.9
Body	12	21.4
Tail	10	17.9
Head and body	6	10.7
Body and tail	4	7.1
The measures of diagnosis		
CT-guided biopsy	20	35.7
Ultrasound-guided puncture	10	17.9
Endoscopic ultrasonography	20	35.7
Clinical N stage		
0	36	64.3
1	20	35.7
Tumor size		
≤ 4 m	16	28.6
>4 cm	40	71.4
Total bilirubin (µmol/l)		
≤ 21	29	51.8
21–100	27	48.2
Direct bilirubin (µmol/l)		
≤ 10	29	51.8
10–80	27	48.2
Pain score		
No pain (0)	14	25.0
Mild and moderate (1–5)	32	57.1
Severe (6–10)	10	17.9
CA19-9 (IU/ml)		
0–37	5	8.9
37–100	20	35.7
> 100	31	55.4

### Dosimetric index

The isodose level of prescription dose in the treatment plan was 75–90%, with a median value of 80%. The irradiation fields involved 150–200 non-coplanar fields. The treatment plan showed that the mean CI of pancreatic lesions in all patients was 1.12, and the mean nCI was 1.27, as shown in Table 2.

**Table 2:** The dosimetry index of the 56 pancreatic cancer patients during CyberKnife radiosurgery treatment

Item	CI	nCI	Coverage (%)	PTV (cm^3^)	Prescription dose (Gy)	Prescription isodose line (%)
Range	1.04–1.27	1.18–1.46	84–96	35–152	30–50	75–90
Mean	1.12	1.27	89	54	42	80
Median	1.15	1.32	90	62	40	82

Coverage: the coverage is volume of the tumor receiving greater than or equal to the prescription dose divided by the total volume of the tumor times 100

*CI* conformity index: the conformity index is the ratio of the tissue volume receiving the prescription isodose or more to the tumor volume receiving the prescription isodose or more

*nCI* new conformity index: the new conformity is the CI multiplied by the ratio of the total tumor volume to the tumor volume receiving the prescription isodose or more

### Local control rate

Out of the 56 patients, five (5/56, 8.9%) achieved complete response, 32 (32/56, 57.1%) exhibited partial response, 15 (15/56, 26.8%) showed stable disease, and four developed progressive disease. The 6-month and 12-month local control rates were 93% and 85%, respectively. Six months after the Cyberknife SBRT, 25 (59.5%) of the 42 patients with abdominal pain showed considerable relief. Weight was increased in five patients, and ECOG PS improved in 15 patients.

### Long-term survival and prognostic factors

**Fig. 1:**
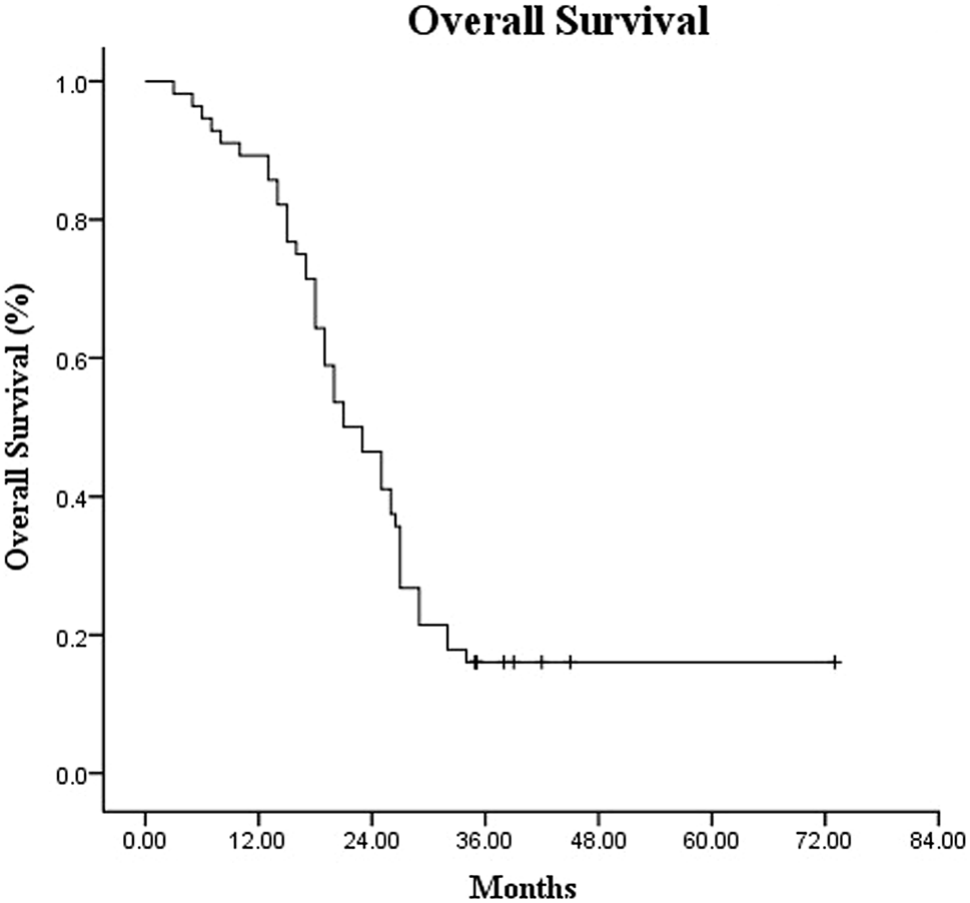
Kaplan–Meier analysis of overall survival for patients with LAPC

**Fig. 2:**
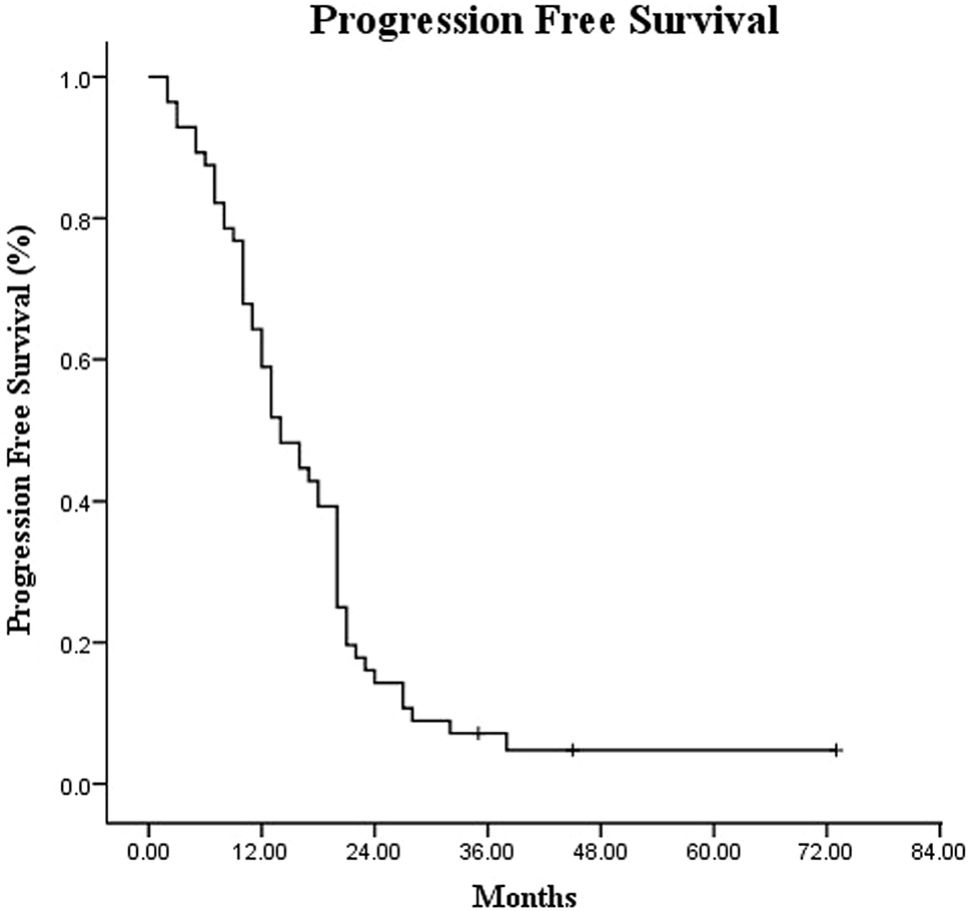
Kaplan–Meier analysis of progression free survival for patients with LAPC

The median follow-up time was 17 months (range 3–43 months). Among the 56 patients, the median OS and PFS times were 19.0 months (95% CI 14.60–23.40) and 12.0 months (95% CI 8.34–15.66), respectively (Figs. 1, 2). The 1-year and 2-year OS rates were 82.1% and 35.7%, whereas the 1-year and 2- year PFS rates were 48.2% and 14.3%, respectively (Figs. 1, 2). Multivariate analysis showed that patients with a tumor diameter of < 4 cm, without lymph node metastasis, with low CA19-9 level before SBRT, and with normal CA19-9 levels after SBRT showed remarkably longer OS (Table 3).

**Table 3:** Prognostic factors for overall survival of (LAPC)

Variable	*N*	Univariate			Multivariate		
		Median	95% CI	*p*	HR	95% CI	*p*
Gender							
Male	29	23.0	16.88–29.12	0.806	1		0.351
Female	27	21.0	17.31–22.69		0.689	0.32–1.51	
Age							
> 60	28	23.0	17.83–28.17	0.629	1		0.501
≤ 60	28	20.0	12.76–27.24		0.772	0.36–1.64	
ECOG							
0	10	27.0	26.24–27.76	0.016	1		0.990
1	38	23.0	16.96–29.04		0.956	0.21–4.44	
2	8	15.0	6.68–23.31		1.042	0.39–2.81	
Tumor location							
Head	30	21	12.28–29.72	0.680	1		0.620
Body	12	23	17.34–28.66		1.646	0.60–4.49	
Tail	14	19	15.33–22.67		1.262	0.44–3.58	
N stage							
0	36	27	26.42–27.58	< 0.0001	1		0.012
1	20	15	13.57–16.43		0.191	0.05–0.70	
Tumor diameter							
> 4 cm	40	26.5	24.95–28.05	< 0.0001	1		< 0.0001
≤ 4 cm	16	13.0	9.08–16.92		0.034	0.006–0.19	
Pain score							
0	14	26	23.56–28.45	0.230	1		0.751
1–5	32	20	16.67–23.33		1.198	0.33–4.36	
6–10	10	18	13.35–22.65		0.819	0.31–2.18	
Pre-SBRT CA19-9 (IU/ml)							
≤ 37	4	21	16.6–25.4	< 0.0001	1		0.026
37–100	20	29	26.14–31.86		0.029	0.002–0.48	
≥ 100	32	17	15.15–18.85		0.184	0.044–0.77	
BED							
≤ 60 Gy	34	18	15.72–20.28	0.019	1		0.400
> 60 Gy	22	26	24.16–27.84		0.681	0.29–1.67	
Post-SBRT CA19-9							
Elevated	7	7	4.43–9.57	< 0.0001	1		0.001
Decreased to normal	28	19	17.29–20.71		67.410	7.45–610.267	
Decreased not to normal	21	23	24.52–39.48		8.505	1.95–37.12	

### CA19-9 determined OS

**Fig. 3:**
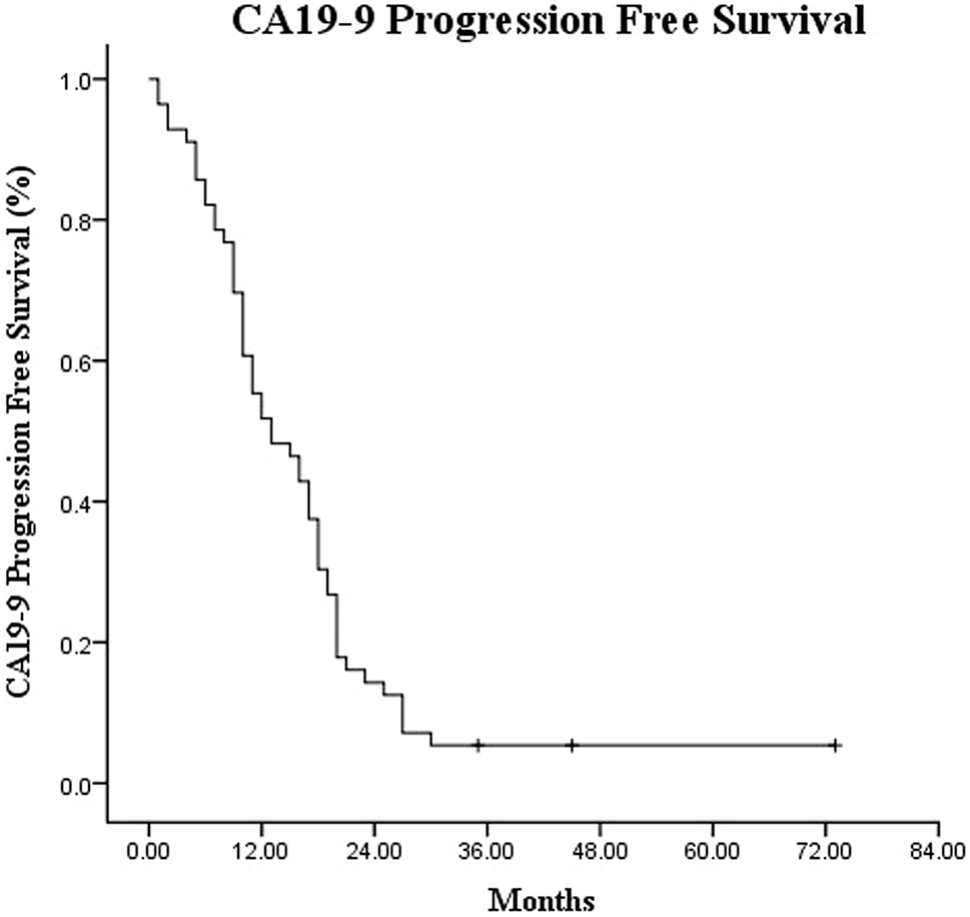
Kaplan–Meier analysis of carbohydrate antigen 19-9-progression-free survival for patients with LAPC

**Fig. 4:**
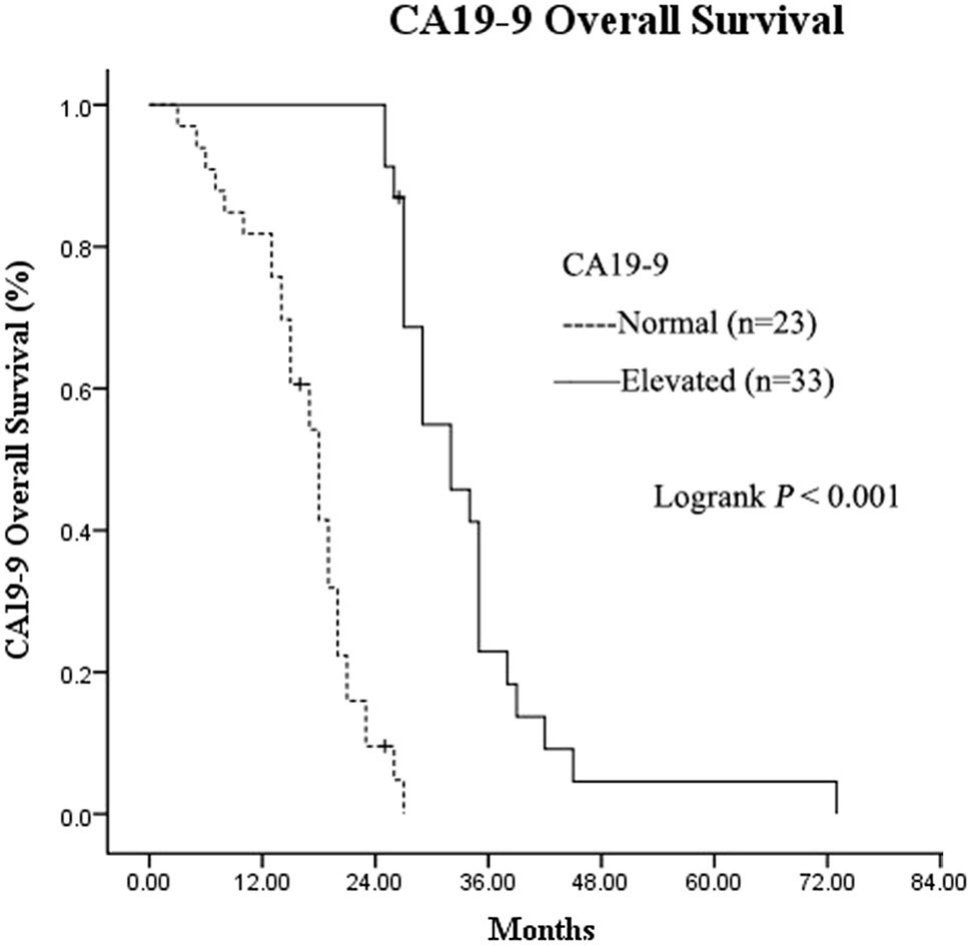
Kaplan–Meier overall survival curves: CA19-9 < 37 IU/ml (*n* = 23, solid lines) versus CA19-9 > 37 IU/ml (*n* = 33, dashed lines) patients with LAPC (*p* < 0.001)

Out of the 56 patients, 51 showed elevated pre-SBRT CA19-9. Among the 51 patients, the median CA19-9 level prior to SBRT, which was 1294 IU/ml (58–8587 IU/ml), decreased to 550 IU/ml (17–3587 IU/ml) 6 months after Cyberknife SBRT. The CA19-9 levels patients normalized in 18 (35.3%) patients, but did not normalize in 27 (52.9%) patients, 24 of which showed 50% reduction. Furthermore, six (11.8%) patients showed elevated CA19-9. The CA19-9-determined PFS was 11.0 months (95% CI 5.77–16.24, Fig. 3). The median OS of post-SBRT CA19-9 normalization, either normal before SBRT or normal after SBRT treatment, was 30 months (95% CI 24.69–35.31), which was remarkably higher than that of patients with elevated post-SBRT CA19-9 with a median OS of 16 months (95% CI 13.33–18.67, Fig. 4).

### Side effects

In acute settings, patients generally experienced grades 1 or 2 fatigue, nausea, and appetite loss. Moreover, 6 cases (10.7%) exhibited acute grade 2 fatigue and 13 (23%) showed grades 2–3 nausea and vomiting, which required additional anti-nausea treatment. Only two (3.5%) patients suffered from grade 3 abdominal pain caused by duodenal stenosis, which was maintained for 1 month. No other acute grade 3 gastrointestinal toxicity and treatment-related deaths were observed. Late toxicity was evaluated > 3 months after SBRT. One patient experienced late grade 4 radiation enteritis, which required urgent operative intervention. One patient showed late grade 3 bleeding caused by gastric ulcer, which necessitated gastroscopic intervention. Two patients suffered from late grade 3 duodenal ulcer, which needed endoscopic treatment. One patient demonstrated a late grade 4 intestinal fistula, which required medical treatment combined with surgical treatment (Table 4)

**Table 4:** Side effects in 56 patents with pancreatic carcinoma in the treatment of CyberKnife SBRT

Category	CTCAE 4.0						
	Total grade ≥ 2 (%)	Total grade ≥ 3 (%)	Grade 1 (%)	Grade 2 (%)	Grade 3 (%)	Grade 4 (%)	Grade 5 (%)
Acute toxicity							
Non-hematologic							
Enteritis	4 (7.1)	0 (0)	12 (21.3)	4 (7.1)	0 (0)	0 (0)	0 (0)
Intestinal fistula	0 (0)	0 (0)	0 (0)	0 (0)	0 (0)	0 (0)	0 (0)
Gastritis	2 (3.6)	0 (0)	15 (26.8)	2 (3.6)	0 (0)	0 (0)	0 (0)
Stomach ulcer	2 (3.6)	0 (0)	3 (5.4))	2 (3.6)	0 (0)	0 (0)	0 (0)
Other GI toxicity							
Nausea	3 (5.4)	0 (0)	4 (7.1)	3 (5.4)	0 (0)	0 (0)	0 (0)
Vomiting	10 (17.9)	2 (3.6)	6 (10.7)	8 (14.3)	2 (3.6)	0 (0)	0 (0)
Weak	6 (10.7)	0 (0)	23 (41.1)	6 (10.7)	0 (0)	0 (0)	0 (0)
Anorexia	7 (12.5)	0 (0)	20 (35.7)	7 (12.5)	0 (0)	0 (0)	0 (0)
Abdominal pain	4 (7.2)	2 (3.6)	8 (14.3)	2 (3.6)	2 (3.6)	0 (0)	0 (0)
Constipation	4 (7.1)	0 (0)	12 (21.4)	4 (7.1)	0 (0)	0 (0)	0 (0)
Bloating	5 (8.9)	2 (3.6)	10 (17.9)	3 (5.4)	2 (3.6)	0 (0)	0 (0)
Hematologic							
Neutropenia	18 (32.1)	6 (10.7)	34 (60.7)	12 (21.4)	6 (10.7)	0 (0)	0 (0)
Thrombocytopenia	12 (21.4)	4 (7.1)	20 (35.7)	8 (14.3)	4 (7.1)	0 (0)	0 (0)
Hemoglobin	11 (19.6)	2 (3.6)	18 (32.1)	9 (16.1)	2 (3.6)	0 (0)	0 (0)
Lymphopenia	5 (8.9)	1 (1.8)	25 (44.6)	4 (7.1)	1 (1.8)	0 (0)	0 (0)
Late toxicity							
Enteritis	2 (3.6)	1 (1.8)	5 (8.9)	1 (1.8)	0 (0)	1 (1.8)	0 (0)
Intestinal fistula	1 (1.8)	1 (1.8)	0 (0)	0 (0)	0 (0)	1 (1.8)	0 (0)
Gastritis	0 (0)	0 (0)	6 (10.7)	0 (0)	0 (0)	0 (0)	0 (0)
Stomach ulcer	1 (1.8)	1 (1.8)	0 (0)	0 (0)	1 (1.8)	0 (0)	0 (0)
Duodenal ulcer	2 (3.6)	2 (3.6)	0 (0)	0 (0)	2 (3.6)	0 (0)	0 (0)

## Discussion

Prognosis in patients with non-metastatic unresectable LAPC is worsened by inoperability. Chemotherapy alone can reduce the rate of distant metastasis in patients with localized disease. The median survival time is reached in 8-15 months, but may barely improve local disease control (Poplin et al. 2009; Kindler et al. 2010). Local progression adversely affects the quality of life and may lead to duodenal obstruction, abdominal pain, bleeding, and obstructive jaundice, which remarkably reduce the quality of life of patients with LAPC.

The combination of radiotherapy and chemotherapy is another treatment method for LAPC (version 1. 2019) and its application in LAPC has always been controversial due to the long duration of conventional radiotherapy and the high toxicity (Hammel et al. 2016; Chauffert et al. 2008; Huguet et al. 2007; Huang et al. 2014). The appearance of SBRT therapeutic modalities compensates for the deficiencies of conventional radiotherapy. Nowadays, SBRT is increasingly applied to LAPC. Zhong et al. compared 7819 patients with LAPC treated with conventional radiotherapy with 631 patients treated with SBRT (Zhong et al. 2017). Multivariate analysis showed that SBRT treatment improved OS (*p* < 0.001). Within the propensity matched cohorts, the median OS (13.9 months vs. 11.6 months) and the 2-year OS rate (21.7% vs. 16.5%) were significantly higher with SBRT than those in conventional radiotherapy (*p* = 0.0014). In another clinical study (Park et al. 2017), 44 patients with inoperable pancreatic cancer were treated with SBRT, whereas 226 patients were treated with intensity modulated radiation therapy (IMRT). Although no remarkable differences were observed in OS, local control rate and distant metastasis rate, grade 2 gastrointestinal toxicity, grade 2 fatigue, and grade 3 hematological toxicity in the SBRT group were remarkably lower than those in the IMRT group. Cheng et al. (2018) developed a model to predict surgical resectability in patients with borderline resectable (BR) and LAPC who undergo SBRT. The median total dose was 33 Gy. A total of 191 patients was identified (128 patients with LA and 63 with BR), of which 87 patients (46%) underwent margin-negative resection. Importantly, radiation dose was a key predictor of resectability in certain subpopulations and the model showed improved accuracy in the prediction of margin-negative resection compared with NCCN guideline staging (75% vs. 63%; *p* < 0.05). SBRT is a minimally invasive treatment option that can be delivered in 1–5 days, in contrast with the approximately 6 weeks of daily treatments for conventional radiotherapy for patients with LAPC. The shortened duration of treatment can remarkably improve the quality of life, the tolerance of treatment for LAPC patients, and improve prediction of surgical resectability.

Most clinical trials for LAPC have involved chemoradiotherapy followed by chemotherapy (Gastrointestinal Tumor Study Group 1988; Klaassen et al. 1985). However, the results obtained are unsatisfactory, mainly because many patients develop distant metastasis during the first few months following the diagnosis and the beginning of treatment. Subjecting patients with distant metastasis to radiation therapy of up to 6 weeks is controversial. Approximately 30–35% of patients who receive neoadjuvant therapy will develop distant metastasis before local treatment (Huguet et al. 2007; Krishnan et al. 2007). Emerging data suggest that providing systemic therapy followed by consolidation chemoradiation in patients who do not demonstrate progression may be preferable in initial chemoradiation (Huguet et al. 2007; Krishnan et al. 2007; Crane et al. 2009). A large retrospective analysis from Crane et al. (2006) found that patients who receive chemotherapy followed by consolidation chemoradiation show longer median survival (11.9 months) than those who receive initial chemoradiation (8.4 months). Ko et al. (2007) also confirmed the aforementioned treatment method, showing median OS reaching 17 months. Although SBRT has been used in LAPC for a short time and the number of the cases is small, most clinical studies adopt chemotherapy followed by SBRT. In general, two to four cycles of induction chemotherapy are followed by SBRT treatment. However, reports on reassessing patients after two to four cycles of chemotherapy are limited. Harvard University Cancer Center (Mahadevan et al. 2011) found that 47 patients with LAPC have been evaluated after two cycles of gemcitabine chemotherapy, eight of which showed distant metastases, and the remaining 39 received SBRT. Furthermore, the median OS was 20 months, and the median PFS reached 15 months, which were higher than those obtained for most studies conducting SBRT for LAPC (Herman et al. 2015; De Bari et al. 2016; Jung et al. 2019).

In our previous study, SBRT alone was used for LAPC without induction chemotherapy. Although SBRT was well tolerated, no adverse events greater than grade 3 occurred, and the annual control rate was only 53.1% (Shen et al. 2010). Moningi et al. (2015) analyzed 88 patients with pancreatic cancer, 74 of which suffered from with LAPC and 14 patients showed critically borderline resectable pancreatic cancer (BRPC). Most of the patients received chemotherapy before SBRT. The median OS and PFS from the date of diagnosis were 18.4 months (LAPC 18.4 months; BRPC 14.4 months) and 9.8 months (95% CI 8.0–12.3), respectively. The 1-year and 2-year OS rates were 60% and 15%, respectively. The 1-year survival rate was higher than that in our previous data, which may be related to the non-employment of induction chemotherapy. Herman et al. (2015) demonstrated that in 49 patients who received SBRT followed by gemcitabine monotherapy, the median OS was 13.9 months (95% CI 10.2–16.7). Freedom from local disease progression at 1 year was 78%. The 1-year and 2-year OS rates were 59% and 18%, respectively, and the median PFS was 7.8 months (95% CI 5.8–10.2). Adverse reactions could be tolerated, but the survival rate was lower than that in the previous clinical study, given that two-thirds of enrolled patients were aged > 65 years, thereby resulting in a decreased survival rate. Mazzola et al. (2018) studied 33 patients with LAPC using Linac-based stereotactic body radiation therapy. Among the patients, 24 received pre-SBRT induction chemotherapy, with a 1-year local control rate of 81% and a 1-year OS rate of 75%, which was similar to previous studies on patients who received induction chemotherapy.

In the present study, 65 patients with LAPC were treated with SBRT after two cycles of induction chemotherapy and reassessment. A total of 56 patients without distant metastasis was treated with SBRT, with a median OS of 19 months, median PFS of 12 months, 1-year OS of 82.1%, and 2-year OS of 35.7%. All median OS and PFS were more favorable than those obtained in the above studies, because we excluded patients with distant metastasis after two cycles of induction chemotherapy. The chemotherapy regimen we adopted was capecitabine combined with gemcitabine, which may also be a factor for the improved results. Cunningham et al. (2009) evaluated whether the addition of capecitabine (CAP) and gemcitabine (GEM) would improve survival over GEM alone in patients with advanced pancreatic cancer. GEM-CAP significantly improved objective response rate (19.1% vs. 12.4%; *p* = 0.034) and PFS (*p* = 0.004) and was associated with a trend toward improved OS (*p* = 0.08) compared with GEM alone. Although GEM-CAP treatment led to increased neutropenia, this phenomenon did not result in febrile neutropenia. The incidences of other side effects were comparable between the two arms. ESPAC-4 clinical trial (Neoptolemos et al. 2017) showed that the median OS for patients with resected pancreatic cancer in the gemcitabine plus capecitabine group was 28.0 months (95% CI 23.5–31.5) compared with the 25.5 months (22.7–27.9) in the gemcitabine group (*p* = 0.032) with no considerable difference in toxic and adverse reactions. Quan et al. (2018) evaluated the efficacy and safety of induction chemotherapy (gemcitabine/capecitabine) followed by stereotactic ablative radiotherapy (SABR) in patient with BR and locally advanced (LA) pancreatic ductal adenocarcinoma (PDAC). The results suggest excellent tolerability, high R0 resection rates, and acceptable posttreatment complications. Therefore, in the present study, we used capecitabine combined with gemcitabine for two cycles of chemotherapy, followed by SBRT treatment after evaluation. We achieved better treatment effect than that reported in most previous studies.

There are also some studies that have achieved better survival results. Krishnan et al. (2016) reviewed the outcomes of LAPC patients treated with dose-escalated intensity modulated radiation therapy (IMRT). Patients who received biologically effective dose (BED) > 70 Gy had a superior OS (17.8 vs. 15.0 months; *p* = 0.03), with estimated OS rates at 2 years of 36% versus 19% and at 3 years of 31% versus 9% along with improved local–regional RFS (10.2 vs. 6.2 months; *p* = 0.05) as compared with those receiving BED ≤ 70 Gy. Patients who received BED > 70 Gy had a similar OS to us. But the IMRT technology, requiring 5–6 weeks, prolonged treatment and reduced patient compliance. In a phase 2 clinical trial, Murphy et al. (2019) evaluated the margin-negative (R0) resection rate of neoadjuvant FOLFIRINOX and losartan followed by chemoradiotherapy for LAPC. Overall median PFS was 17.5 months and median OS was 31.4 months. Among patients who underwent resection, median PFS was 21.3 months and median OS was 33.0 months. The median OS and PFS were all superior to our results. The main reason was that most patients completed neoadjuvant FOLFIRINOX for eight cycles, and then most patients completed surgical treatment. FOLFIRINOX regimen does have a good effect, but is not suitable for Asian patients due to the high toxicity. Rudra et al. (2019) reviewed 44 patients with inoperable pancreatic cancer treated with adaptive magnetic resonance imaging (MRI)‐guided radiation therapy (MRgRT). In their results, high-dose patients had statistically significant improvement in 2-year OS (49% vs 30%; *p* = 0.03) compared to standard-dose patients. The results were similar to us. MRgRT is a novel modality that potentially allows for dose escalation, while minimizing excessive radiation dose to the organs at risk (OAR). There may be some technical advantages in MRgRT and we can also explore them in the future.

CA19-9 is the most sensitive tumor marker for pancreatic cancer. CA19-9 offers important clinical value in the diagnosis of pancreatic cancer, evaluation the efficacy of radiotherapy and chemotherapy, prognosis evaluation, and OS judgment. Schellenberg et al. (2008) evaluated the efficacy of a single fraction of 25 Gy SBRT delivered between cycles 1 and 2 of gemcitabine chemotherapy. The median survival for those with normal and elevated CA19-9 levels was 12.7 and 9.6 months, respectively (*p* = 0.09). Furthermore, the survival of patients with normal CA19-9 level, either at diagnosis or 6 weeks after SBRT, was remarkably improved compared with that of patients whose CA19-9 failed to normalize after treatment. The median survival for patients who achieved normal and constantly elevated CA19-9 levels was 13.3 and 7.3 months, respectively (*p* < 0.01). Gurka et al. (2017) studied 38 patients with inoperable pancreatic cancer using SBRT combined with chemotherapy. The median OS was 14.3 months and the median PFS was 9.2 months. Before SBRT treatment, the PFS with baseline CA19-9 level that was lower than the median was significantly higher than the baseline CA19-9 level that was higher than the median (*p* = 0.0002). However, the results were not convinced in other research. Stanford University Medical Center reported 77 patients with inoperable pancreatic cancer who received SBRT, 45 of which suffered from LAPC (Chang et al. 2009). Univariate analysis indicated that initial CA19-9 level (normal vs. elevated) and percent drop in CA19-9 (from initial to nadir) did not influence OS or freedom from local progression (FFLP). In the present study, the median CA 19-9-determined PFS time was 11 months (95% CI 5.77–16.24). The median OS of post-SBRT CA19-9 normalization (whether normal before treatment or normal after SBRT treatment) was 30 months (95% CI 24.69–35.31), which was remarkably higher than that of patients with elevated post-SBRT CA19-9 with a median OS of 16 months (95% CI 13.33–18.67). Multivariate analysis demonstrated that pre-SBRT CA19-9 level and post-SBRT CA19-9 declines were independent prognostic factors (*p* < 0.05). Monitoring CA19-9 level during treatment is crucial in evaluating prognosis.

In the current study, four serious intestinal late toxicity, (one grade 4 intestinal fistula, two grade 3 late duodenal ulcer, and one grade 4 enteritis) were all occurred in patients with the lesions located in pancreatic head, which were adjacent to the duodenum. While most of the patients had just grade 1/2 adverse effects. As a result, we concluded that SBRT was safe for most of pancreatic patients. We further analyzed the four intestinal patients with serious adverse effects. The total dose in part of the duodenum reached 35 Gy, which maybe exceeded the duodenum tolerance, although the irradiation volume was small. No adverse reactions > grade 3 were found in acute toxicity. Gastrointestinal complications after SBRT for pancreatic cancer commonly occur (Moningi et al. 2015; Chuong et al. 2013). Methods to provide an improved segmentation model and limit the dose of normal tissue remain to be explored, especially given that lesions are adjacent to the stomach and duodenum. Feng et al. (2018) and Rao et al. (2017) assessed the feasibility and theoretical dosimetric advantages of an injectable hydrogel to increase the space between the head of the pancreas (HOP) and duodenum. They demonstrated the feasibility of hydrogel separation of the HOP and duodenum might improve the outcomes and reduce the duodenal dose in patients with unresectable pancreatic cancer before SBRT. In the future, we will select patients who might benefit from hydrogel placement and to predict the required hydrogel spacing to achieve clinical constraints. In our study, prophylactic proton pump inhibitors have been used in all patients, excluding those who showed direct expansion of the tumor to the intestine or stomach during endoscopy, which may contribute to the toxicity of SBRT. Given that pancreatic tumors move with breathing, CyberKnife SBRT avoids the errors caused by respiratory movement through synchronous respiratory tracking technology. Therefore, the technology can avoid normal tissue, such as stomach and duodenum, surrounding tumor lesions (Yang et al. 2014).

Although the role of SBRT in LAPC patients has not been fully established, our single-institution experience suggests that SBRT results in minimal toxicity and improved survival based on the history reports of patients treated with conventional CRT. Cyberknife SBRT can be delivered safely and quickly to potentially benefit patients with locally advanced unresectable pancreatic cancer. However, additional prospective studies are necessary to determine how SBRT can be optimally integrated with systemic chemotherapy regimens to improve PFS, OS, and patients’ quality of life compared with conventional chemoradiotherapy.

## Electronic supplementary material

Below is the link to the electronic supplementary material.

Supplementary material 1 (DOCX 15 kb)
