# Supplementary material for: Clinical outcomes and prognostic factors of stereotactic body radiation therapy combined with gemcitabine plus capecitabine for locally advanced unresectable pancreatic cancer
Source: J Cancer Res Clin Oncol. 2019 Oct 30;146(2):417–28. doi: 10.1007/s00432-019-03066-z (PMC11804411; doi:10.1007/s00432-019-03066-z)
Supplement: Supplementary file 1 — Supplementary material 1 (DOCX 15 kb) [file 432_2019_3066_MOESM1_ESM.docx]

**Table 1 The standard of dose limitation in critical structures**

| Critical structures | Dose type  Mean(range) | 5 fractions | |
| --- | --- | --- | --- |
|  |  | Volume (cc) | Dose (Gy) |
| stomach | Max point^a^ dose | Any point | <32 |
|  | Volume* | <10cc | 18 |
| duodenum | Max point^a^ dose | Any point | <32 |
|  | Volume* | <5cc | 18 |
|  | Volume* | <10cc | 12.5 |
| Jejunum/ileum | Max point^a^ dose | Any point | <35 |
|  | Volume* | <5cc | 19.5 |
| spinal cord | Max point^a^ dose | Any point | <30 |
|  | Volume* | <0.35cc | 23 |
|  | Volume* | <1.2cc | 14.5 |
| Liver | Volume^#^ | >700cc | 21 |
| Left/Right-kindey | Volume^#^ | >200cc | 17.5 |
| Renal hilum/vascular trunk | Volume (%) | 66% | <23 |

a: “Point” defined as 0.035 cc or less

*: Max critical volume above threshold

#: Minimum critical volume below threshold
